# Muscle mass, not radiodensity, predicts physical function in cancer patients with or without cachexia

**DOI:** 10.18632/oncotarget.27594

**Published:** 2020-05-19

**Authors:** Lindsey J. Anderson, Nicole Chong, Dorota Migula, Adam Sauer, Michelle Garrison, Peter Wu, Atreya Dash, Jose M. Garcia

**Affiliations:** ^1^Geriatric Research, Education and Clinical Center, Veterans Affairs Puget Sound Health Care System, Seattle, Washington, USA; ^2^Department of Surgery, Veterans Affairs Puget Sound Health Care System, Seattle, Washington, USA; ^3^Department of Health Services, University of Washington, Seattle, Washington, USA; ^4^Department of Surgery, University of Washington, Seattle, Washington, USA; ^5^Department of Urology, Veterans Affairs Puget Sound Health Care System, Seattle, Washington, USA; ^6^Department of Urology, University of Washington, Seattle, Washington, USA; ^7^Gerontology and Geriatric Medicine-Department of Medicine, University of Washington, Seattle, Washington, USA

**Keywords:** cancer cachexia, physical function, computed tomography, muscle radiodensity

## Abstract

Background: There is a need to better understand the relationship between functional impairment and muscle mass in cancer cachexia. This study aimed to establish the relationship between computed tomography (CT)-derived muscle cross-sectional area (CSA), radiodensity, and skeletal muscle index (SMI) and dual energy X-ray absorptiometry (DXA) parameters with functional performance in cancer patients.

Materials and Methods: Handgrip strength, stair climb power (SCP), one-repetition maximum (1RM) strength, and body composition (CT and DXA) were compared across cancer patients with cachexia (CAC; *N* = 28), without cachexia (CNC; *N* = 28), and non-cancer patients (CON; *N* = 19). Multivariate regression was performed to find predictors of function.

Results: CAC had lower CT muscle CSA and SMI and lower DXA appendicular lean mass (ALM) than CNC or CON (*p* ≤ 0.011). Muscle radiodensity was not different across groups despite larger proportion of low CT SMI in CAC, and CAC performed worse in SCP than CON (*p* = 0.018). In cancer patients, DXA ALM and CT muscle CSA each predicted physical function (*p* ≤ 0.05); muscle radiodensity did not, and DXA ALM explained more variability in SCP and 1RM than CT muscle CSA.

Conclusions: Stair climb power was reduced in cancer cachexia; muscle radiodensity was not. Muscle mass by CT or DXA, but not radiodensity, predicted functional performance in cancer patients.

## INTRODUCTION

Cancer cachexia (CAC) is a complex metabolic syndrome characterized by involuntary loss of muscle, with or without loss of fat, leading to functional impairment [1]. Cachexia is experienced by up to 74% of all cancer patients, with the highest incidences occurring in gastrointestinal (GI), genitourinary (GU), lung, and head and neck cancers [2]. Cachexia has a negative impact on chemotherapy tolerance [3], functional status, quality of life [4, 5], and survival [6] being responsible for up to 22% of cancer-related deaths [7].

There are no current treatments approved by the U. S. Food and Drug Administration for CAC [8, 9] and multiple phase III clinical trials completed recently have failed to elicit clinically meaningful improvements in physical function in spite of causing an increase in lean body mass [10–13]. For example, treatment with agents such as anamorelin or enobosarm have shown improvements in handgrip strength (HGS) or stair climb power (SCP), respectively, in phase II trials [14, 15], but these functional outcomes were not improved in phase III trials when testing these agents [10, 13, 16]. However, while HGS and SCP were not responsive to these respective treatments, it is not known whether other aspects of physical function that were not captured may have been impacted in those trials. Due to the complexity of CAC, there is a pressing need to improve our understanding of functional impairment in CAC to better inform methodology, intervention goals, and endpoints in future clinical trials.

Computed tomography (CT) and dual energy X-ray absorptiometry (DXA) can be used to measure body composition in the cancer setting [17]. It is recommended that assessment of muscle mass is essential to cachexia assessment, especially when making comparisons between weight-losing cancer patients and weight stable cancer or non-cancer control patients [18]. Assessment of muscle mass by DXA is commonly used due to the relative ease of analysis compared to CT; however, advantages of CT analysis over DXA is the ability to assess 1) skeletal muscle directly in contrast with DXA assessment where lean mass is derived from measurements of total body mass, fat mass, and bone mass and also 2) radiodensity (SMD) via muscle attenuation (Hounsfield Units, HU), which is thought to be reduced by fatty infiltration [19]. Hence, assessment of muscle depletion and radiodensity by CT is emerging as a promising tool in the cancer setting [20–22]. Additionally, some reports suggest an association between CT-derived SMD or skeletal muscle index (SMI; muscle area corrected for stature) and objective [23–25] or self-reported physical function [26] in cancer patients but this relationship is not well-established.

Therefore, the aim of this study was to assess the relationship between CT-derived muscle cross-sectional area (CSA), SMI, and SMD or DXA-derived appendicular lean mass (ALM) and appendicular skeletal muscle index (ASMI) with objective physical function in cancer patients with or without cachexia, and in non-cancer, age-matched controls. We hypothesized that 1) CT measures (CSA, SMI, and SMD), DXA measures (ALM and ASMI) and physical function would be significantly reduced in patients with cancer cachexia compared to cancer patients without cachexia and to non-cancer control patients, 2) CT measures (CSA, SMI, and SMD) and DXA measures (ALM and ASMI) would be significant predictors of physical function in cancer patients with or without cachexia, and 3) that SMD would be correlated with muscle mass by different measures.

## RESULTS

Demographic information for cancer patients with cachexia (CAC, *N* = 28), cancer patients without cachexia (CNC, *N* = 28), and non-cancer, weight-stable control patients (CON, *N* = 19) is provided in Table 1. Compared to CNC and CON, CAC had significantly lower body weight and BMI and greater 6-month relative weight change (*p* < 0.001); CNC had a significantly higher proportion of stage 1 tumors than CAC (Table 1). Five (9%) of the total 56 cancer patients were undergoing active chemotherapy and/or radiotherapy.

**Table 1:** Participant descriptives

Med (SEM) or *N* (%)	CAC *N* = 28	CNC *N* = 28	CON *N* = 19	*p*-value
**Age (yrs)**	67.5 (1.7)	66.0 (1.7)	64.0 (1.9)	0.51
**Height (cm)**	177.2 (1.7)	177.8 (1.6)	177.8 (1.5)	0.93
**Weight (kg)**	77.5 (4.0)	96.4 (3.7)^a^	95.9 (3.4)^a^	**< 0.001**
**BMI (kg/m^2^)**	24.5 (1.1)	31.5 (0.9)^a^	27.9 (1.1)^a^	**< 0.001**
**6-mo weight change (%)**	-8.5 (1.2)	-0.3 (0.5)^a^	-2.9 (0.7)^a^	**< 0.001**
**Ethnicity**				0.26
White, non-Hispanic	22 (78.6)	21 (75.0)	13 (68.4)	
White, Hispanic	2 (7.1)	0 (0.0)	1 (5.3)	
Black/African American	3 (10.7)	1 (3.6)	4 (21.1)	
Asian/Pacific Islander	1 (3.6)	3 (10.7)	0 (0.0)	
Native American	0 (0.0)	1 (3.6)	0 (0.0)	
Unknown/Declined	0 (0.0)	2 (7.1)	1 (5.3)	
**Tumor system**				1.00
Gastrointestinal	19 (67.9)	19 (67.9)	—	
Genitourinary	9 (32.1)	9 (32.1)	—	
**Tumor stage**				**0.009**
1	3 (10.7)	14 (50.0)^a^	—	
2	11 (39.3)	6 (21.4)	—	
3	7 (25.0)	6 (21.4)	—	
4	7 (25.0)	2 (7.1)	—	
**Recent treatment^b^**				
Chemotherapy (y)	10 (35.7)	5 (17.9)	—	0.23
Radiotherapy (y)	5 (17.9)	3 (10.7)	—	0.71

^a^vs CAC; ^b^within 3-months prior to study visit. CAC, cancer with cachexia; CNC, cancer no cachexia; CON, control; y, yes.

Muscle CSA was significantly lower in CAC than CNC or CON (*p* < 0.001, Table 2). There was a trend for a difference across groups in proportion of patients with low SMI (*p* = 0.093, Table 2). Muscle radiodensity was not different across groups when assessed altogether (*p* = 0.76, Table 2) or separately for images with contrast-enhancement (*p* = 0.72, data not shown) or without contrast (*p* = 0.96, data not shown). The proportion of CT scans performed with contrast-enhancement and the number of days between CT capture and study visit were not different across groups. Weight change between CT capture and study visit was greater for CAC than CNC (*p* = 0.005, Table 2). CAC displayed lower DXA measures of LBM, ALM, and ASMI and greater proportion of participants with low ASMI than CNC or CON (*p* ≤ 0.011, Table 2).

**Table 2:** Body composition assessed at the lumbar (CT) or whole-body (DXA) level

Med (SEM), *N* (%)	CAC *N* = 28	CNC *N* = 28	CON *N* = 19	*p*-value
**Computed tomography**				
Muscle CSA (cm^2^)	145.5 (5.9)	177.7 (5.6)^a^	175.5 (6.4)^a^	**< 0.001**
Muscle radiodensity (HU)	35.8 (1.4)	34.3 (1.6)	36.8 (1.8)	0.76
CT with contrast (y)	20 (71.4)	21 (75.0)	16 (84.2)	1.00
SMI (cm^2^/m^2^)	44.3 (1.9)	54.8 (1.6)^a^	55.0 (2.4)	**< 0.001**
Low SMI (y)	16 (57.1)	8 (28.6)	8 (42.1)	0.093
Days: CT to study visit (d)	-30.5 (8.3)	-27.0 (8.3)	27.0 (40.9)	0.61
Wt change: CT to study visit (%)	-3.0 (0.6)	0.0 (0.4)^a^	-0.1 (1.0)^b^	**0.005**
**Dual Energy X-ray Absorptiometry**	**CAC *N* = 14**	**CNC *N* = 14**	**CON *N* = 10**	
LBM (kg)	56.3 (4.0)	72.1 (3.0)^a^	70.7 (3.3)^a^	**0.002**
ALM (kg)	23.1 (1.6)	28.7 (1.3)^a^	30.4 (1.7)^a^	**0.011**
ASMI (cm^2^/m^2^)	6.7 (0.4)	8.9 (0.3)^a^	9.4 (0.5)^a^	**0.001**
Low ASMI (y)	9 (32.1)	0 (0.0)^a^	1 (10.0)^a^	**< 0.001**

^a^vs CAC; ^b^vs CAC (*p* = 0.065); CAC, cancer with cachexia; CNC, cancer no cachexia; CON, control; CSA, cross-sectional area; HU, Houndsfield Units; SMI, skeletal muscle index; LBM, total lean body mass; ALM, appendicular lean mass; ASMI, appendicular SMI; y, yes.

**Figure 1:**
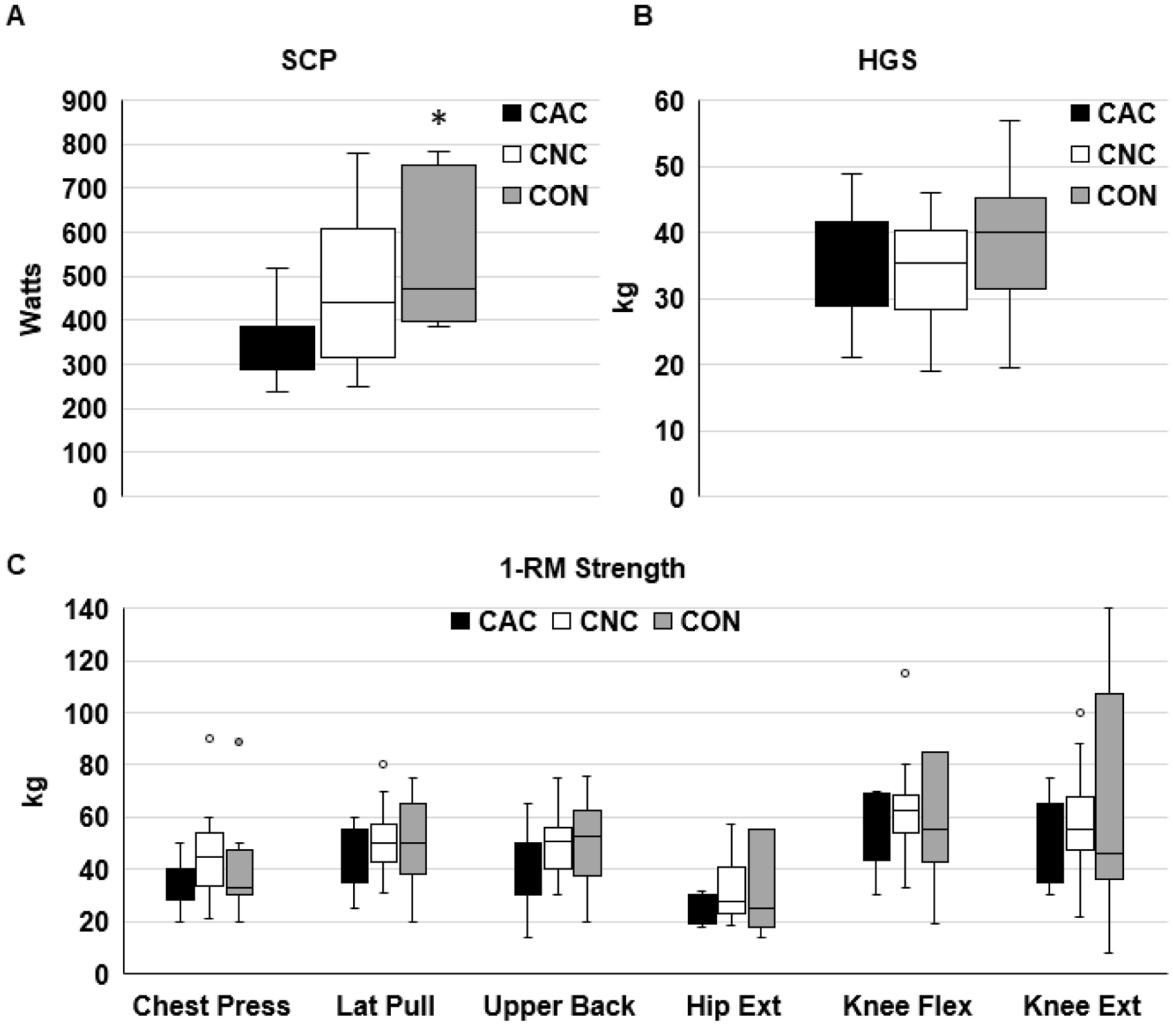
Physical function in patients with or without cachexia and controls. ^*^ *p* ≤ 0.05 vs CAC. Stair climb power ((**A**): CAC, *n* = 12; CNC, *n* = 12; CON, *n* = 7), handgrip strength ((**B**): CAC, *n* = 23; CNC, *n* = 24; CON, *n* = 17), and 1-repetition maximal strength ((**C**): CAC, *n* = 9–12; CNC, *n* = 12–14; CON, *n* = 7–8).

**Figure 2:**
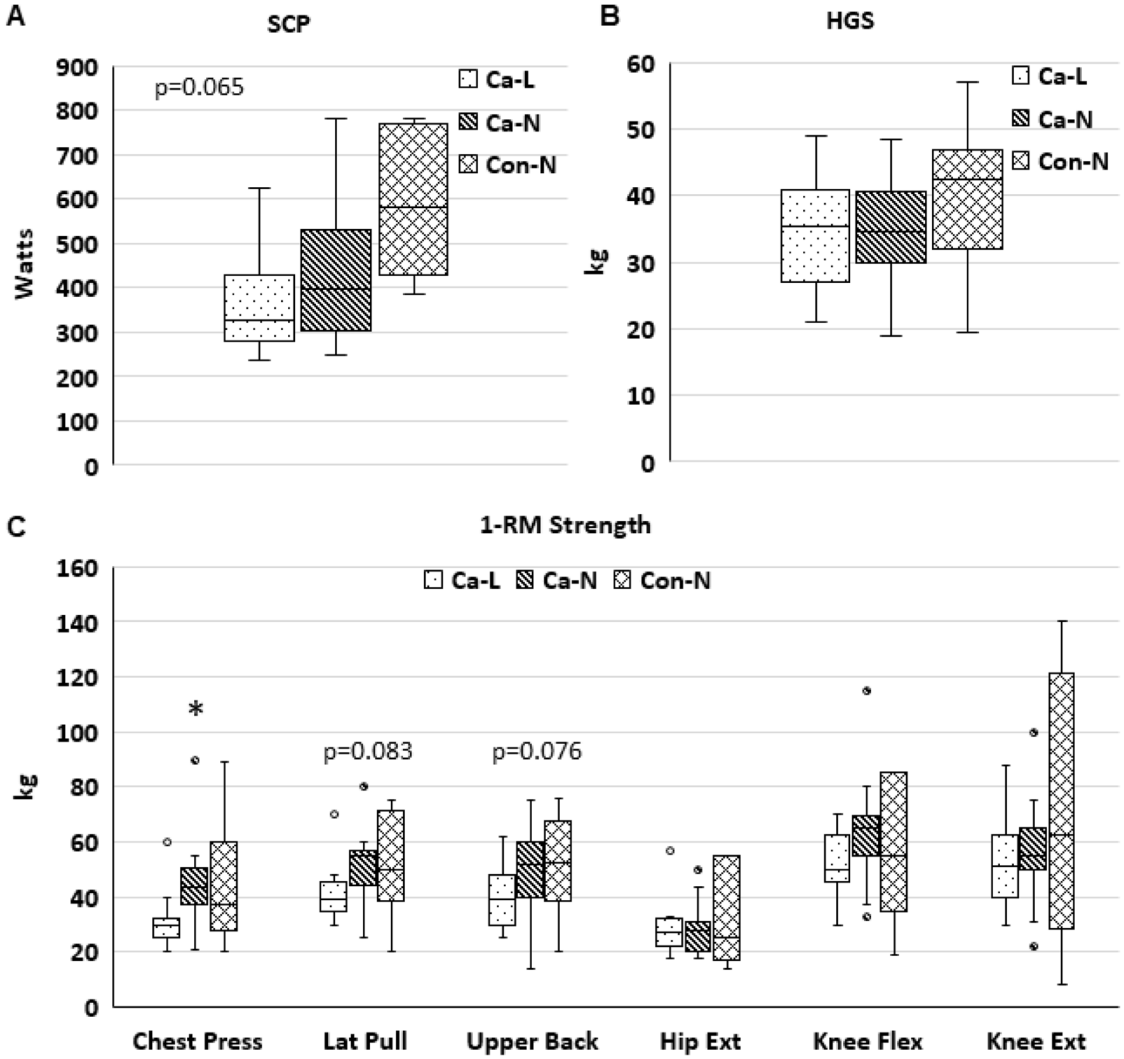
Physical function in patients with low or normal CT-SMI. ^*^ *p* ≤ 0.05 vs Ca-L. Stair climb power ((**A**): Ca-L, *n* = 9; Ca-N, *n* = 15; Con-N, *n* = 5), handgrip strength ((**B**): Ca-L, *n* = 21; Ca-N, *n* = 27; Con-N, *n* = 10), and 1-repetition maximal strength ((**C**): Ca-L, *n* = 9–10; Ca-N, *n* = 12–16; Con-N, *n* = 5–6).

Physical function measured by SCP was significantly worse in CAC than CON (*p* = 0.018, Figure 1A). There were no differences across groups for HGS or 1-repetition maximum (1RM) muscle strength, except for a trend for a difference across groups in Chest Press 1RM (*p* = 0.095, Figure 1B and 1C). On an exploratory analysis we also investigated physical function after patients were stratified by cancer and muscle index by CT into three groups: cancer with low SMI (Ca-L, *N* = 24), cancer with normal SMI (Ca-N, *N* = 32), and non-cancer controls with normal SMI (Con-N, *N* = 11, Figure 2A–2C). The non-cancer controls with low SMI were excluded from this exploratory analysis due to the lower number of functional assessments completed in these patients. Chest Press 1RM was lower in Ca-L than in Ca-N (Figure 2C); a trend for a difference across groups was seen in SCP (*p* = 0.065) and 1RM for Lat Pull (*p* = 0.083) and Upper Back (*p* = 0.076; Figure 2A, 2C). There were no differences across the three groups for HGS or lower body 1RM. SMD was significantly lower in Ca-L than Ca-N (*p* = 0.026) and Con-N (*p* = 0.029), data not shown. Cachexia incidence was almost twice as great in Ca-L (66.7%) than Ca-N (37.5%; *p* = 0.058). Age was significantly greater in Ca-L than Ca-N (*p* = 0.021) or Con-N (*p* = 0.013).

Multivariate regression was completed for different models using either variables for DXA or for CT (Table 3). Each model contained the following conditional variables: age, BMI, tumor system, stage, and 6-month relative weight change. Each model additionally contained either: 1) DXA conditional variables (ALM, ASMI), or 2) CT conditional variables (SMI, muscle CSA, SMD). Significant predictors with unstandardized Beta and 95% confidence intervals for each dependent variable (physical function parameter) are provided in Table 3.

**Table 3:** Prediction of physical function by models including either DXA or CT parameters in cancer patients

Dependent Variable	Models including DXA variables				Models including CT variables			
	N (R^2^)	Predictor (s)	Unstd. B (95% CI)	*p*-value	N (R^2^)	Predictor (s)	Unstd. B (95% CI)	*p*-value
**HGS (kg)**	27	None	n/a	n/a	45 (0.19)	Muscle CSA	0.08 (0.02–0.14)	0.017
						Tumor	4.64 (0.21–9.07)	0.046
**SCP (W)**	23 (0.51)	ALM	9.42 (0.60–18.24)	0.05	23 (0.28)	Muscle CSA	2.52 (0.80–4.24)	0.009
		Age	-8.59 (–14.25– –2.93)	0.008				
		%Wt change	10.62 (1.74–19.50)	0.03				
**Chest Press (kg)**	24 (0.72)	ALM	1.38 (0.77–1.99)	< 0.001	24 (0.56)	Muscle CSA	0.31 (0.19–0.43)	< 0.001
		Age	–0.88 (–1.33– –0.43)	0.001				
		%Wt change	0.96 (0.23–1.69)	0.016				
**Lat Pull (kg)**	24 (0.50)	ALM	1.50 (0.85–2.15)	< 0.001	24 (0.55)	Muscle CSA	0.29 (0.17–0.41)	< .0001
**Upper Back (kg)**	25 (0.65)	ALM	1.79 (1.24–2.34)	< 0.001	25 (0.58)	Muscle CSA	0.28 (0.18–0.38)	< 0.001
**Hip Extension (kg)**	21 (0.24)	BMI	0.80 (0.17–1.43)	0.023	21 (0.24)	BMI	0.80 (0.17–1.43)	0.023
**Knee Flexion (kg)**	26 (0.58)	ALM	3.00 (1.78–4.22)	< 0.001	26 (0.35)	Muscle CSA	0.28 (0.12–0.44)	0.002
		BMI	–1.14 (–2.20– –0.08)	0.047				
**Knee Extension (kg)**	24 (0.21)	ALM	1.49 (0.29–2.69)	0.024	24	None	n/a	n/a

DXA, dual energy X-ray absorptiometry; CT, computed tomography; N, sample size, R^2^, model R-squared, Unstd. B, unstandardized Beta; CI, confidence interval; HGS, handgrip strength; SCP, stair climb power; ALM, appendicular lean mass; Wt, body weight; CSA, cross-sectional area; SMI, skeletal muscle index; BMI, body mass index.

The predominant predictor of physical function in the models containing DXA variables was ALM, displaying a positive relationship with all outcomes except HGS and Hip Extension 1RM (Table 3). Greater age and greater weight loss were associated with worse SCP and Chest Press strength, in addition to ALM, in the models containing DXA variables. In the models containing CT variables, muscle CSA was the predominant predictor of physical function displaying a positive relationship with all outcomes except Hip Extension and Knee Flexion 1RM (Table 3). Hip Extension 1RM was predicted by BMI, but not by either DXA or CT variables.

DXA-derived ALM, in combination with age and 6-month relative weight change, explained more variability in SCP and Chest Press 1RM and ALM alone explained more variability in Upper Back 1RM than CT-derived muscle CSA (Table 3). In combination with BMI, ALM explained more variability in Knee Flexion 1RM than CT-derived muscle CSA (Table 3).

CT measures of muscle CSA were positively correlated with DXA measures of lean mass (*p* ≤ 0.05, Table 4); SMD was not correlated with any DXA lean mass variables. CT muscle radiodensity was positively correlated with CT muscle CSA (*r* = 0.33, *p* = 0.014).

**Table 4:** Spearman correlations between DXA and CT in cancer patients

Correlation Coefficients ®, *N* = 28	CT: CSA (cm^2^)	CT: SMD (HU)	CT: SMI (cm^2^/m^2^)
** DXA: LBM (kg)**	0.87^a^	–0.05	0.66^a^
** DXA: ALM (kg)**	0.84^a^	–0.02	0.62^a^
** DXA: ASMI (kg/m^2^)**	0.85^a^	–0.07	0.80^a^

^a^ *p* ≤ 0.05, DXA, dual energy X-ray absorptiometry; CT, computed tomography; LBM, lean body mass; ALM, appendicular lean mass; ASMI, appendicular skeletal muscle index; CSA, cross-sectional area; HU, Hounsfield Units.

## DISCUSSION

In this cohort of patients with GI or GU cancer, cachexia was associated with reduced muscle mass but not with reduced muscle radiodensity. Muscle mass was a significant predictor of physical function, but muscle radiodensity was not. Patients with cancer cachexia had lower SMI and worse functional performance than controls as measured by SCP, and when stratified according to muscularity, cancer patients with low SMI exhibited decreased chest press strength compared to those with normal SMI. The relationship between physical function and muscle mass was observed regardless of whether body composition was assessed via CT or DXA; however, DXA models generally explained more variability in physical function than CT models.

Analysis of standard-of-care CT images is gaining traction as a valuable tool for studying body composition as it may reduce patient burden and cost of research procedures and provide more information when compared to DXA scans [1, 27]. However, DXA is commonly reported in the literature with widely available reference data for comparison. Yet the relationship between muscle mass, muscle radiodensity, and physical function is still not well-known. While some studies have shown an association between different functional tests and SMI [24], others have not [25]. In the current study, we observed that muscle mass as measured by CT-obtained muscle CSA was a positive predictor of most functional outcomes, whereas SMI and SMD did not significantly predict function. The discrepancy between studies may be due to differences in sample size, tumor type, functional outcome measures, or other demographic characteristics such as gender and race.

Despite prior reports showing an association between LBM and HGS [15], no DXA parameters significantly predicted HGS in our study. Instead, a small amount of variability in HGS was explained by CT muscle CSA, suggesting that while HGS may be better predicted by CT muscle CSA, HGS may not be an optimal outcome for assessment of physical function in this setting. Also, this observation may explain why Phase III clinical trials assessing muscle mass via DXA and physical function via HGS, fail to show a concomitant improvement in muscle mass and function.

DXA ALM, a surrogate measure for muscle mass [28], was a better predictor than CT muscle CSA for SCP and strength. Overall, ALM was a better predictor of upper body strength than CT-derived muscle CSA; although, ALM explained a similar proportion of Lat Pull strength than muscle CSA. Similarly, ALM was a better predictor of leg strength than CT-derived muscle CSA, excluding hip extension which was only predicted by BMI. The greatest amount of variability explained by any model was the prediction of Chest Press strength by DXA ALM. DXA models explained at least 50% of variability in the prediction of SCP, all three upper body strength assessments, and knee flexion, whereas CT models only explained at least 50% of variability in the prediction of the three upper body strength assessments.

In healthy older adults, changes in SMD explained a larger portion of variability of changes in muscle strength than changes in muscle mass [29, 30]. While the current study design is cross-sectional, we did not observe this relationship between SMD and physical function. Other studies have reported a relationship between greater SMD and better physical function in cancer patients as measured by the Timed Up and Go test [25], HGS [23], or gait speed [23]. This disagreement may be due to high prevalence (50–75%) of active cancer treatment in those two other cohorts [23, 25] and low prevalence (9%) in the current study. In addition, neither of these two previous reports addressed the issue of CT image contrast-enhancement in their analyses or results. As the use of CT-derived SMD emerges as a factor of interest in physical function assessment, a consensus is needed for reporting CT parameters and appropriate statistical methodology to account for the impact of image contrast on SMD. In the current study, SMD was not different across groups when analyzed separately for unenhanced images or enhanced images, suggesting that radiodensity was not impacted by cachexia in this cohort.

While the difference in number of patients with low SMI across groups in the current study did not reach significance, this number was over twice as high in cancer patients with cachexia (57%) than cancer patients without cachexia (28%). Using the same SMI cut-point, low SMI was reportedly 21.6% in a cohort of male patients with GI cancer, similar to that observed here for cancer patients without cachexia [23]. However, 24% of that cohort met the weight loss criteria for cachexia, indicating that their reported prevalence of low SMI is likely lower than in our study. This is the first study to compare low muscularity between CT (low SMI) and DXA (low ASMI) in cancer patients or older adults. We observed greater number of patients with low muscularity from CT than DXA analyses for all three study groups. Low muscularity was almost twice as prevalent from CT than DXA for cancer patients with cachexia, while DXA did not identify anyone in the non-cachectic cancer group, and only one in the control group, with low ASMI.

When patients were stratified by CT-derived muscularity, the only difference in functional performance observed here was reduced chest press strength in cancer patients with low SMI compared to cancer patients with normal SMI. In contrast to Barbalho and colleagues, we did not observe a difference in HGS across groups based on SMI stratification [23]. Considering that the current cohort displayed roughly twice the HGS as the Barbalho et al. male cohort, the discrepancy may be because most of that cohort were undergoing chemotherapy and/or radiotherapy at the time of functional assessment. However, the stratification used by those authors was based on SMI and SMD, whereas we did not stratify by SMD in the current study due to the combination of contrast-enhanced and non-contrast images utilized here. This issue was not addressed by Barbalho et al.; however, contrast-enhancement significantly increases SMD and reduces identification of myosteatosis, or low SMD [23, 31]. In another report using the same SMI cut-points, there were no differences in functional impairment for activities of daily living between cancer patients with low or normal SMI [25], suggesting that SMI may not be especially important for quality of life in carrying out daily functions.

Additionally, there is a lack of consensus regarding parameters for CT-based evaluation of sarcopenia. We used CT SMI cut-points defined by Martin and colleagues to stratify patients into low and normal SMI groups; these threshold values were established based on prognostic value in the context of muscle depletion in obesity [21]. Although other studies have also used the same cut-points in this setting [23–25], there is still no well-established criteria [32]. In addition, parameters used for tissue segmentation vary among studies [33–35]. We chose threshold values based on what has been more consistently used in other studies, but there continues to be a need to standardize CT parameters for tissue segmentation. Our findings suggest that lumbar muscle CSA does predict physical function even though differences physical function were only seen for chest press when stratifying groups by these cut-points. The fact that these cut-points did not display a difference in most functional outcomes could be because they were developed for mortality and are BMI-specific. Unlike the CT cut-points, the DXA cut-points for low ASMI are not BMI specific but are more widely used in the literature for sarcopenia assessment than CT cut-points. However, the predictive value of these cut-points for physical function was not significant for DXA-derived ASMI.

This study has many strengths including utilization and comparison of commonly used body composition estimation tools, inclusion of cancer patients with or without cachexia in addition to age-matched patients without cancer, assessment of multiple objective functional parameters, and uniform distribution of tumor types and recent treatment exposure between cancer patients with or without cachexia. This study is limited by the lack of female patients, lack of case-matching for tumor stage, single L3 slice analysis, missing data for sub-analyses, and its cross-sectional design that prevents us from establishing causation. In addition, the functional tests utilized here are, in some cases, not directly testing the muscles assessed by the body composition tools but are commonly reported in cancer and other sarcopenic populations [14, 15, 36–38]. The quadriceps musculature may be considered most important for functional ability, but this is not assessed by CT here, and there is minimal mid-thigh CT reference data available for comparison. We have included SCP and lower body 1-RM as measures of quadriceps function even though quadriceps musculature is only assessed by DXA ALM in the current study. In addition, as L3 muscle CSA is highly correlated with total LBM [39, 40], it may be reasonable to hypothesize that it would be associated with physical function assessed by tests that don’t directly target the abdominal muscles.

With the urgent need to understand functional impairment in the context of cancer cachexia, we report that SCP and upper body strength were negatively impacted by cachexia and low SMI. We also report that muscle mass assessed by CT or DXA, but not CT-derived SMD, were associated with physical function and that DXA ALM explained greater variability in physical function outcomes in patients with GI or GU cancer. It remains to be confirmed on a larger scale whether the relationship between muscle mass or radiodensity and physical function are each confined to specific functional outcomes and whether they are generalizable to various tumor types.

## MATERIALS AND METHODS

### Design and subjects

This was a single-center, cross-sectional observational study conducted at the Veterans Affairs Puget Sound Health Care System (VAPSHCS) in Seattle, WA, USA. This protocol was approved by the VAPSHCS Institutional Review Board and the Research and Development Committee and was conducted in compliance with the Declarations of Helsinki and its amendments and the International Conference on Harmonization Guideline for Good Clinical Practices.

Males with histologically, cytologically, or image-based documented GI or GU cancer were recruited from oncology or urology clinics at VAPSHCS. Weight-stable (no weight loss > 5% in the prior six months; body mass index (BMI) < 20 kg/m^2^ with weight loss > 2%; or weight loss > 2% with sarcopenia) males with no history of active cancer (except for non-melanoma skin cancer) within the last five years were recruited as controls from general surgery or urology clinics at VAPSHCS. Participants were excluded if they had other conditions associated with cachexia (e. g. congestive heart failure, liver disease, renal failure); active, uncontrolled infection; uncontrolled diabetes mellitus (defined as HbA1c ≥ 9%); actively using an anabolic or investigational agent; or did not have a clinically available CT scan at the third lumbar (L3) level. Control patients had a clinically available CT scan as part of the evaluation for the following conditions: incisional hernia, benign prostatic hyperplasia, adenomatous colon polyp, gallstones, colonic diverticuli, or desmoid-type fibromatosis.

Cancer cachexia was defined by any of the following criteria: involuntary weight loss > 5% in the prior six months; body mass index (BMI) < 20 kg/m^2^ with weight loss > 2%; or weight loss > 2% with sarcopenia [1]. Sarcopenia (low skeletal muscle index, SMI) was defined as appendicular skeletal muscle index (ASMI) < 7.0 kg/m^2^ measured by DXA or lumbar SMI < 43 cm^2^/m^2^ (males with BMI less than 25 kg/m^2^) or < 53 cm^2^/m^2^ (males with BMI ≥ 25 kg/m^2^) measured by CT [21, 32].

### Study visit

Participants reported to the VAPSHCS after a night of fasting. Body composition was measured for lean body mass (LBM), appendicular lean mass (ALM), and ASMI (ALM [kg] / height [m^2^]) by DXA (Hologic Inc., Marlborough, MA) [41]. Objective physical function was measured by HGS (Jamar Hydraulic Dynamometer, J. A. Preston Corp., Clifton, NJ), SCP, and 1RM muscle strength. Stair climbing power was measured by having participants climb a flight of standard hospital stairs (13 steps, 15.3 cm each) at the highest speed safely possible according to their capabilities [42]; up to three trials were attempted, where the shortest time was recorded and used to calculate power: Watts (W) = (body mass [kg] × gravitational acceleration [9.81 m/s^2^] × vertical distance [1.99 m])/time (s). Muscle strength was measured according to the American College of Sports Medicine strength testing guidelines [43] for upper body [Chest Press, Latissimus Pull-down (Lat Pull), Upper back seated row (Upper Back)] and lower body [Hip Extension, Knee Flexion (Knee Flexion), and Knee Extension (Knee Extension)] muscle groups (Kaiser Sports Health Equipment, Inc., Fresno, CA).

### CT analysis

Clinically available spiral CT scans involving the L3 level were obtained from patients’ electronic medical record. Cross-sectional area and radiodensity of skeletal muscle (psoas, paraspinals (quadratus lumborum, erector spinae), abdominals (lateral and oblique) and rectus abdominus) were quantified using image analysis sliceOmatic software (v5.0, TomoVision, Montreal, Quebec, Canada) with attenuation parameters –29 to 150 HU [39, 44, 45]. Different researchers (L. A., A. S., N. C.) were trained to correctly identify and quantify lumbar vertebrae and skeletal muscles. L. A. re-analyzed images after analysis by A. S. and N. C.; an intra-observer coefficient of variation of 1.3% was required. Muscle area was normalized for height (m^2^) and reported as lumbar SMI (cm^2^/m^2^). Mean SMD (HU) is reported for the entire muscle area at the L3 vertebra. Slice thickness ranged from 0.6–3.0 mm, tube voltage ranged from 110–120 kilovolts, and all images with contrast were obtained from the venous portal phase.

### Statistical analysis

SPSS version 18 (SPSS, Inc., Chicago, IL) was used for statistical analysis. Comparison across groups was analyzed using Kruskal-Wallis ANOVA for continuous variables or Fisher’s exact test for categorical variables. The primary outcome was CT-derived CSA and the secondary outcome was SCP; all other outcomes were considered exploratory. For muscle CSA, a sample size of 6 subjects per groups was calculated to be sufficient to detect a difference between groups of 20 cm^2^ with an estimated standard deviation of 10 cm^2^, power 0.9, and Type I error probability of 0.05. For SCP, a sample size of 10 subjects per groups was calculated to be sufficient to detect a difference between groups of 150W with an estimated standard deviation of 50W, power 0.9, and Type I error probability of 0.05. Statistical significance was 2-sided, α ≤ 0.05. Data are reported as median (SEM) or N (%). Multivariate regression was used to identify significant predictors of physical function using cancer patient data only. Spearman correlations were used to determine univariate associations between outcomes in cancer patient data only.

## Abbreviations

1RM: one-repetition maximum
ALM: appendicular lean body mass
ASMI: appendicular skeletal muscle index
BMI: body mass index
CAC: patients with cachexia
CT: computed tomography
CON: weight stable, non-cancer control patients
CNC: patients without cachexia
CSA: cross-sectional area
DXA: dual energy X-ray absorptiometry
GI: gastrointestinal
GU: genitourinary
HGS: handgrip strength
HU: Hounsfield Units
LBM: lean body mass
QOL: quality of life
SCP: stair climb power
SMD: skeletal muscle radiodensity
SMI: skeletal muscle index
Ext: Extension
Flex: Flexion
